# How telomere maintenance affects endometriosis development: a preliminary study

**DOI:** 10.7150/ijms.102646

**Published:** 2025-03-24

**Authors:** Xiaoling Zhao, Weimin Kong, Dan Luo, Yunkai Xie, He Zhang

**Affiliations:** 1 Department of Gynecology, Beijing Obstetrics and Gynecology Hospital, Capital Medical University. Beijing Maternal and Child Health Care Hospital, Beijing, China.; 2 Department of Obstetrics and Gynecology, Beijing Tongren Hospital, Capital Medical University, Beijing, China.

**Keywords:** Endometriosis, Hormones, Nomograms, Telomere

## Abstract

**Background:** Endometriosis results in dysmenorrhea, dyspareunia, chronic pelvic pain and infertility in reproductive-age women. However, no effective treatment methods have been applied to the disease, and the pathogenesis of endometriosis is unclear.

**Purpose:** This study was performed to investigate the association between telomere maintenance and endometriosis development.

**Materials and methods:** The telomere length of the postmenopausal endometria, eutopic endometria and their matched ectopic lesions in the proliferative and secretory phases was detected using fluorescence in situ hybridization (FISH) methods, and the effect of telomere length maintenance on the proliferation of endometrial cells derived from endometriotic patients was determined by 3-(4,5-dimethylthiazol-2-yl)-2,5-diphenyltetrazolium bromide (MTT) assay with BIBR1532 treatment. Then all of the telomere maintenance genes were extracted from the Telnet database, and bioinformatics analysis was performed to uncover the role of telomere maintenance genes in endometriosis development.

**Results:** Telomere length was longer in endometriotic patients' eutopic endometria during the proliferative and secretory phases, and treatment with a telomerase inhibitor inhibited the proliferation of epithelial cells and stromal cells. Furthermore, the telomere maintenance genes were enriched in several hormone-related pathways, with several genes differentially expressed between normal endometria and endometria derived from endometriotic patients. The nomogram constructed based on telomere maintenance genes also displayed good predictive value.

**Conclusions:** Telomere maintenance may contribute to the development of endometriosis, with several related genes involved.

## Introduction

Endometriosis is a chronic disease characterized by the presence of viable endometrial cells outside of the uterus 1, 2. Dysmenorrhea, dyspareunia, chronic pelvic pain and infertility are common symptoms of endometriosis, affecting 10% of reproductive-age women 1, 3. The incidence of endometriosis is 50% in women with infertility and 50-80% in women with pelvic pain 1, 3. Currently, the pathophysiology of endometriosis is not well understood, and treatment protocols are not perfect.

Telomeres are nucleoprotein structures at the end of each chromosome that can maintain genome stability 4, 5. Telomeres shorten as cells divide, thereby resulting in arrested cell growth or cell death when they become too short 4, 5. It is postulated that telomere maintenance is vital for the ectopic survival of endometrial cells. Telomere length is reported to be longer during the secretory phase in the eutopic endometria of women with endometriosis than that in normal endometria 6. Compared with normal endometria, higher levels of telomerase activity and telomerase components, which are helpful for maintaining telomere length, can also be observed in the eutopic endometria of women with endometriosis 6-8. However, the differences in telomere length between eutopic endometria of endometriosis patients and that of control women during proliferative phases remain unclear, and the telomere length changes in ectopic lesions are unknown. Therefore, in this study, we collected samples from endometriosis patients and control women, including normal endometria, eutopic endometria of endometriosis patients and their matched ectopic lesions during the proliferative phase and secretory phase, to explore the variations in telomere length in different tissues. Moreover, cellular experiments were also performed to investigate the roles of telomere maintenance in endometriotic cells.

Telomere maintenance is a complex process involving multiple genes 9, 10. The rapid development of sequencing technology and the emergence of bioinformatics analysis and public databases provide us with the opportunity to obtain a massive amount of gene information. Therefore, in this study, we extracted genes associated with telomere maintenance from the Telnet database and investigated the involvement of telomere maintenance in the development of endometriosis based on datasets derived from the Gene Expression Omnibus (GEO) database, which may improve our understanding of the pathogenesis of endometriosis and provide new targets for endometriosis therapy.

## Methods

### Study population

The samples for telomere length detection were obtained from patients with indications for hysterectomy at Beijing Obstetrics and Gynecology Hospital from January 2018 to December 2021. Normal endometria, including 6 postmenopausal endometria, 6 proliferative endometria and 6 secretory endometria, were collected from patients with grade III cervical intraepithelial neoplasia or stage IA1 cervical cancer. Ectopic lesions and matched eutopic endometrial samples were collected from 6 patients with endometriosis combined with grade III cervical intraepithelial neoplasia or cervical cancer stage IA1. The pathological diagnosis was confirmed postoperatively. All patients had normal menstrual cycles and didn't receive any hormone therapy. Endometrial samples were dated using histological criteria, the date of the last menstrual period and hormone profiles. The samples used in this study were approved by the Beijing Obstetrics and Gynecology Hospital affiliated with the Capital Medical University Ethics Committee (approval number: 2019-KY-002-01). Written informed consent was obtained from each subject, and all experiments conformed to the Declaration of Helsinki.

### Telomere length detection by FISH

Telomere length was measured using the method described by Fishbein 11 with some modifications. The isolated tissues were formalin-fixed and embedded to form paraffin sections. Then the slides were deparaffinized in xylene and rehydrated in a graded ethanol series. The slides were treated for 5 minutes at 37°C with 10 µg/mL proteinase K (Sigma). Slides were probed using a PNA FISH probe (1:100 dilution) (Panagene F1002-5) at 42℃ overnight. Cell nuclei were stained with DAPI (1:750) (Invitrogen) and incubated for 10 min at room temperature away from light. After blocking with anti-fluorescence quenching blocker solution, the sections were observed under a Nikon inverted fluorescence microscope, and images were collected. Mean fluorescence intensity, which is calculated by Image J, was used to quantify the telomere length. Mean fluorescence intensity is equal to the sum of the fluorescence intensities in the region / the area of the region.

### Cell viability assay

Primary endometrial cells were isolated using endometrial samples derived from endometriosis patients of reproductive age according to the method described by Chen 12. The samples obtained in the hospital were preserved in phosphate-buffered saline (PBS) at 4 ℃ and transferred to the laboratory. Then the samples were cut into l mm^3^ pieces and digested with phosphate-buffered saline containing collagenase type I (1 mg/mL, 15 U/mg) and 1% (v/v) penicillin/streptomycin for 1 hour in an orbital shaker at 37°C. Tissue residue was removed by filtration through cell strainers with 100 µm pores (BD Bioscience), and then the stromal cells and epithelial cells were separated through strainers with 40 µm pores (BD Bioscience). Endometrial epithelial cells were collected from the pellet in sieves and cultured in defined keratinocyte serum-free medium (DKSFM; Gibco). The filtrate was centrifuged at 1200 *g* for 5 min, and the supernatant was discarded. Then, endometrial stromal cells were suspended and cultured in DMEM/F12 with 10% fetal bovine serum (FBS; Corning) and 1% (v/v) penicillin/streptomycin.

Endometrial stromal cells were plated and grown in 96-well plates at a concentration of 4000 cells/well for 24 h, while endometrial epithelial cells were plated and grown in 96-well plates at a concentration of 5 clumps/well for 7 days. The cells were subsequently treated with varying doses of BIBR1532 for 48 h. MTT (5 mg/ml) was added to 96-well plates at 5 μl/well, followed by an additional hour of incubation. Then, 100 µl of DMSO was used to terminate the MTT reaction, and the absorbance was read at 490 nm. The effect of BIBR1532 was calculated as a percentage of untreated controls. The experiment was repeated 3 times with cells of different origins.

### Telomere maintenance gene identification

More than 2000 telomere maintenance-related genes derived from humans and yeast are included in the Telnet database (http://www.cancertelsys.org/telnet/), which also assigns each gene a Telnet score to indicate how important it is for telomere maintenance 13. The maximum Telnet score is 10.

### Protein-protein interaction (PPI) network construction and enrichment analysis

The PPI network of the genes was built using stringApp v1.5.1 in Cytoscape software v3.7.1 (https://apps.cytoscape.org), with an interaction score determined as 0.7 14. The clusters were established by using the Molecular Complex Detection (MCODE) plug-in with the default parameters to identify the important modules of the PPI network 15.

CytoHubba, a plug-in of Cytoscape software, was used to rank nodes and screen the hub genes. Because too many genes with the same score were calculated by maximal clique centrality (MCC), EcCentricity and ClusteringCoefficient, only nine topological algorithms (density of maximum neighborhood component [DMNC], maximum neighborhood component [MNC], degree,) were used in this study 16.

The enrichment analysis was also performed by Cytoscape software to screen the pathways in which the genes were involved, including KEGG pathways, wikipathways and reactome pathways, using plug-in string enrichment 14.

### Data collection and DEG identification

The gene expression profiles of GSE51981, GSE120103, GSE37837 and GSE7305 were obtained from the Gene Expression Omnibus (GEO) database.

GSE51981 was produced with the GPL570 platform, containing 34 normal endometria (proliferation phase, n=20; secretory phase, n=14) and 77 endometria from endometriotic patients (proliferation phase, n=29; secretory phase, n=46; unknown=2) 17. Moreover, 49 patients suffered from moderate/severe endometrioma and 28 patients suffered from minimal/mild endometrioma 17.

GSE120103 was produced with the GPL6480 platform, containing 18 normal endometria and 18 endometria from endometriosis patients with stage Ⅳ ovarian endometriosis during the secretory phase 18.

GSE37837 was produced with the GPL6480 platform, containing paired eutopic and ectopic endometrial samples obtained during the proliferative (n=13) and secretory (n=5) phases of the menstrual cycle, among which 8 patients suffered from moderate endometrioma and 10 patients suffered from severe endometrioma 19.

GSE7305 was produced with the GPL570 platform, containing 10 ovarian endometriosis and 10 matched control endometria from the same patients (proliferative phase, n=2; secretory phase, n=8) 20.

### Diagnostic model establishment

The nomogram was created in R using the rms package and the training dataset GSE51981 21. The test dataset, GSE120103, with 36 samples, was used to validate the model. The genes included in the diagnostic model analysis were chosen by the least absolute shrinkage and selection operator (LASSO) regression using the glmnet package 22. The receiver operating characteristic (ROC) curve calculated by the pROC package was used to evaluate the efficacy of the diagnostic model 21.

## Results

### Telomere length differences across eutopic endometria and ectopic lesions

**Figure 1:**
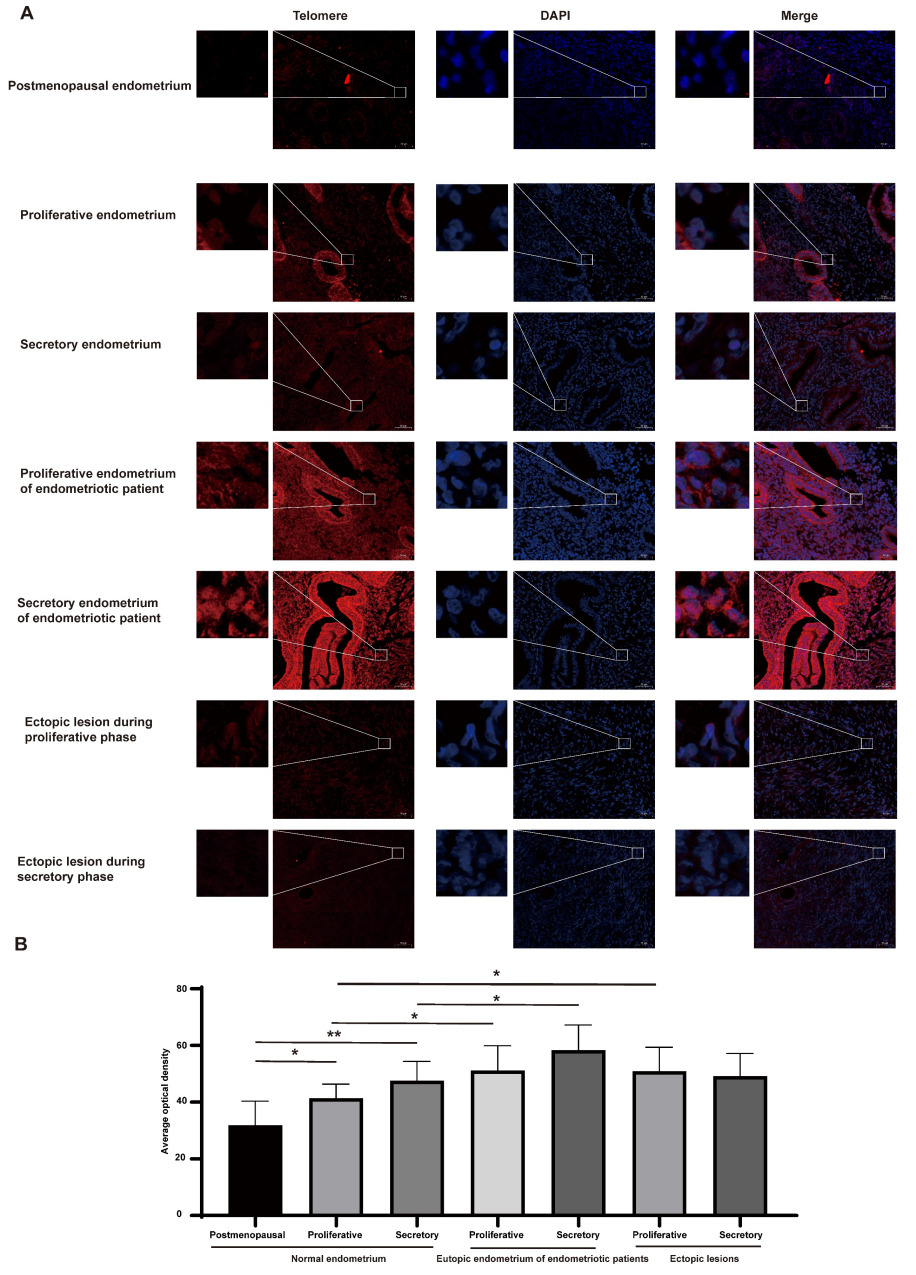
Telomere length examination in different populaitons. (A) Representative images of telomere staining in different populations. (B) The corresponding histograms of positive telomere staining in different populations. Scale bar 50 µm. * p<0.05; ** p<0.01; *** p<0.001; **** p<0.0001.

Telomere length was assessed using FISH methods (Figure 1A-B). Although positive telomere staining was detected in both pre- and postmenopausal endometrial cells, the telomere length of the endometria in postmenopausal women was significantly shorter than that of the premenopausal women (P<0.05). Furthermore, compared to normal endometria during the proliferative and secretory phases, telomere length was longer in endometriotic patients' eutopic endometria (P<0.05). However, there were no significant differences in telomere length between eutopic endometria of endometriosis patients and their matched ectopic lesions. The telomere length in ectopic lesions derived from patients during proliferative phases was longer than that in the normal proliferative endometria (P<0.05), while the telomere length between ectopic lesions derived from patients during secretory phases and normal secretory endometria did not show a significant difference.

### Telomere maintenance affects the proliferation of endometrial cells derived from endometriotic patients

**Figure 2:**
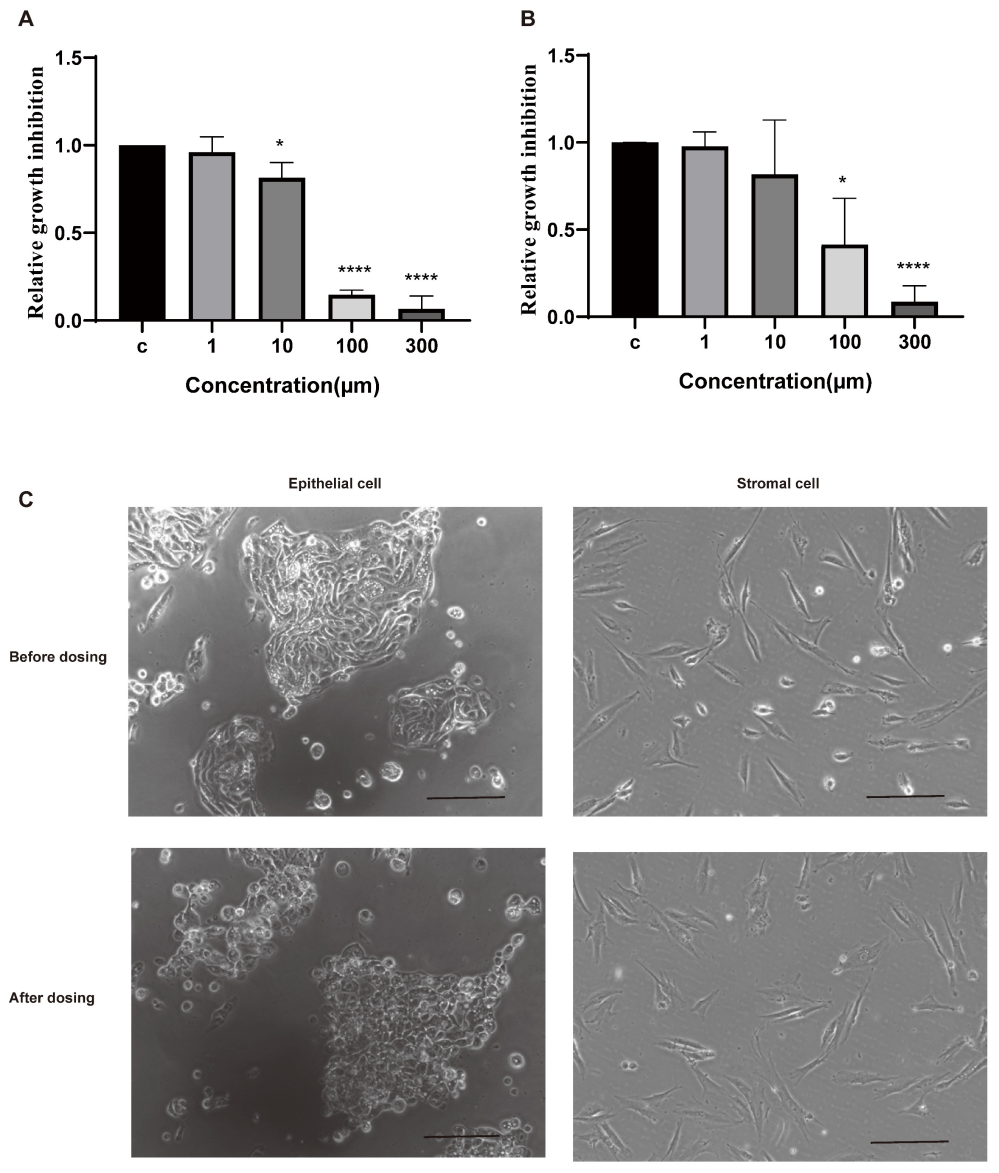
The response of the isolated human endometrial cells to the telomerase inhibitor BIBR1532 after treatment for 48 h. (A-B) Treatment with BIBR1532 for 48 h significantly inhibited the viability of endometrial epithelial cells (A) and stramol cells (B). (C) Representative images of epithelial cells and stromal cells treated with 100 µm BIBR1532 for 48 h. Scale bar 50 µm. * p<0.05; ** p<0.01; *** p<0.001; **** p<0.0001.

Epithelial cells and stromal cells are the main components of the eutopic endometria and ectopic lesions 1. To investigate the roles of telomere maintenance in endometriotic cells, we isolated cells from the eutopic endometria of patients with endometriosis and dosed them with BIBR1532 for 48 h. BIBR1532 is a noncompetitive small-molecule telomerase inhibitor that can prevent telomere lengthening 23. The results showed that the viability of epithelial cells and stromal cells was decreased with the treatment of BIBR1532 for 48 h (Figure 2A-B). Moreover, after treatment with 100 µm BIBR1532 for 48 h, the morphology of the epithelial cells derived from the patients with endometriosis was altered while no visible change was found in stromal cells (Figure 2C).

### PPI network analysis of telomere maintenance genes

**Figure 3:**
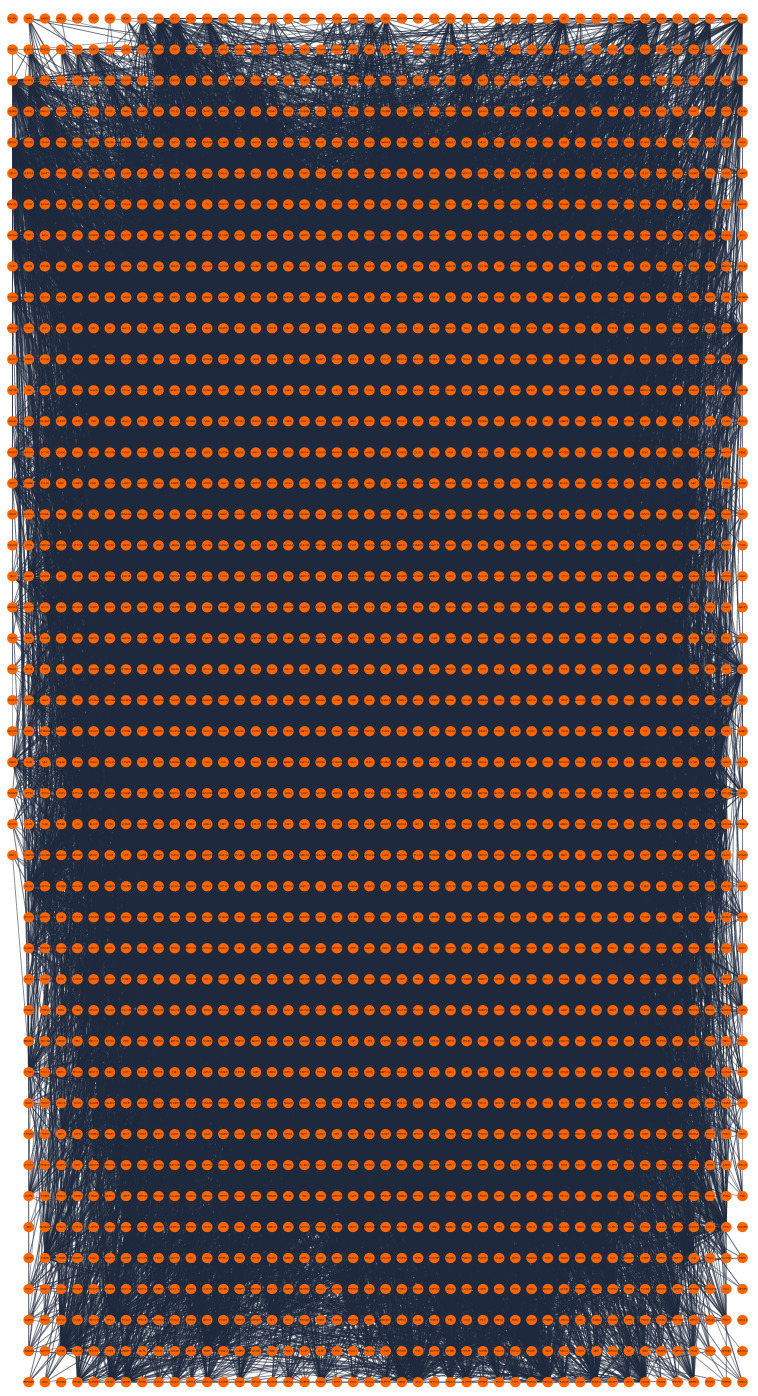
PPI network construction of telomere maintenance genes. PPI, protein-protein interaction.

**Figure 4:**
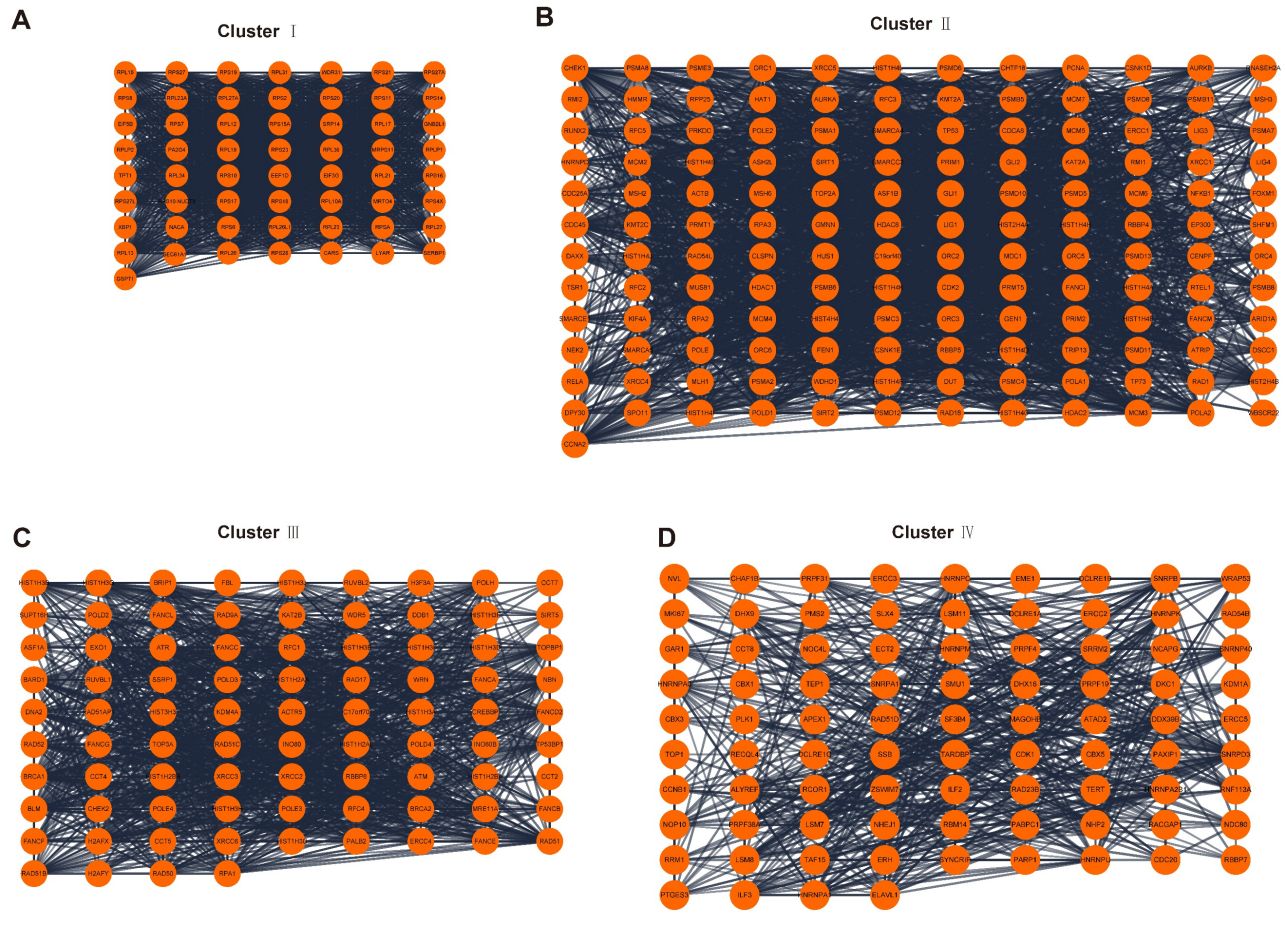
PPI network analysis. (A-D) Four clusters within PPI network scoring above 10. PPI, protein-protein interaction.

A total of 2093 genes were extracted from the Telnet database, and then a PPI network with 2053 nodes and 23727 edges was created (Figure 3). Four clusters within the PPI network scoring above 10 were identified using MCODE (Figure 4A-D), and the information of the clusters was displayed in Table 1.

CytoHubba was used to rank nodes. After the duplicated genes were eliminated, 33 hub genes were selected from the top 10 genes in each method. The significance of the hub genes for telomere maintenance was displayed in Table 2.

### The enrichment analysis of telomere maintenance genes

Endometriosis is a hormone-dependent disease, so we filtered steroid hormone signaling pathways among the enriched pathways. The results showed that there were 5 estrogen-related pathways, 1 progesterone-related pathway, 1 androgen-related pathway and 1 FSH-related pathway, with 133 genes identified (Table 3). The expression profiles of the 133 genes in the GSE51981 and GSE120103 datasets were then investigated. Only 8 genes were significantly differentially expressed, with an adj. p value<0.05 and |log FC|>1 in GSE51981 and GSE120103, among which FOS, FOSB, SRC and MAPK3 were increased in the eutopic endometria of endometriotic patients, while AR, MAPK1 and HSP90B1 were decreased in the eutopic endometria of endometriotic patients (Table 4). Then, the expression of the 133 genes in ectopic lesions was analyzed in datasets GSE37837 and GSE7305. None of them were found to be significantly differentially expressed between ectopic lesions and their matched eutopic endometria derived from the same patients (data not shown). Among the 7 genes differentially expressed between the eutopic endometria of endometriotic patients and normal endometria, only the expression of FOS and FOSB in ectopic lesions was higher than that in eutopic endometria derived from the same patients. However, a significant difference in FOSB and FOS between ectopic lesions and their matched eutopic endometria was observed only in GSE37837 (Table 5). The steroid hormone signaling pathways in which these 7 genes were involved were displayed in Table 6.

### The utility of telomere maintenance genes in the diagnosis of endometriosis

**Figure 5:**
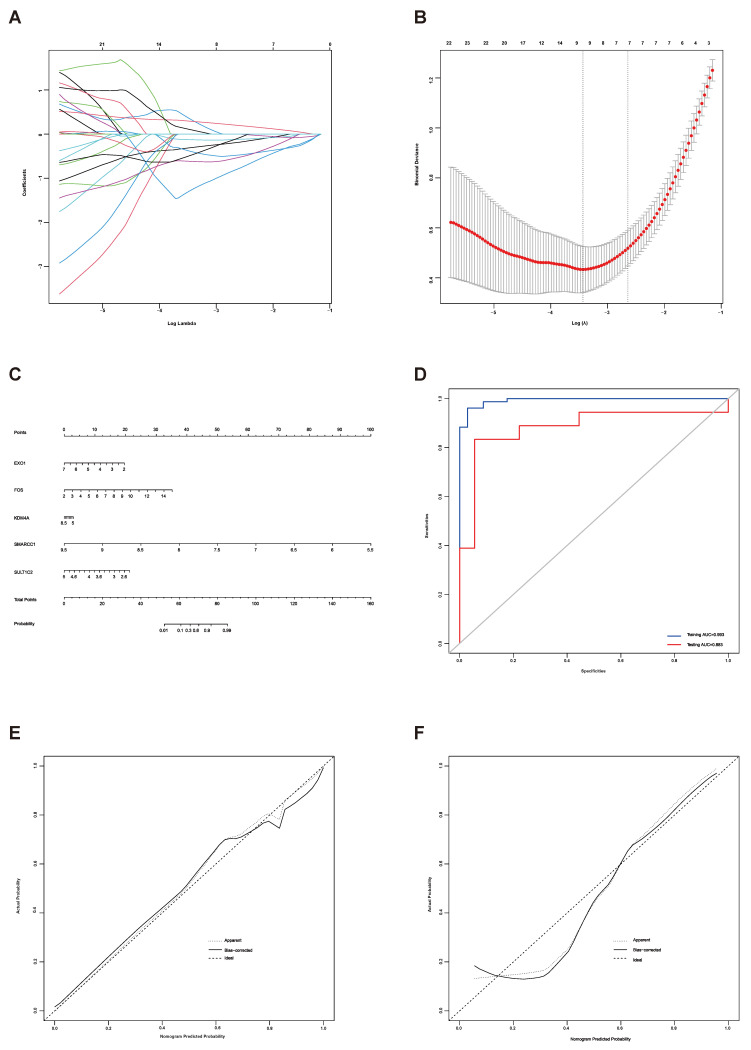
Establishment of a diagnostic model for endometriosis. (A) LASSO coefficient profiles of the genes in the normal endometrium and eutopic endometrium tissue from the endometriosis patients. (B) Selection of the optimal parameter (lambda) in the LASSO model for the normal endometrium and eutopic endometrium tissue from the endometriosis patients. (C) Nomogram model established based on the LASSO regression results. (D) ROC curve of the diagnostic nomogram model for the training dataset (GSE51981) and test dataset (GSE120103). (E-F) The calibration plots for the nomogram based on training dataset (E) and test dataset (F). LASSO, least absolute shrinkage and selection operator; ROC curve, receiver operating characteristic curve.

LASSO regression analysis was used to screen the genes from all the telomere maintenance genes, and EXO1, FOS, KDM4A, SMARCC1, and SULT1C2 were identified (Figure 5A-B). The information and expression of the genes in the four datasets were displayed in Tables 7-8. Then, based on these results, a nomogram model was built (Figure 5C). To test the diagnostic efficacy and the power of the nomogram model, the ROC of the training dataset and test dataset was plotted, yielding an AUC of 0.993 on the training samples and 0.883 on the test samples (Figure 5D). The calibration plots for the training and test datasets were also shown (Figure 5E-F).

## Discussion

Telomeres are nucleoprotein structures found at the ends of chromosomes that contain double-stranded tandem repeats of TTAGGG 4, 5. The main function of telomeres is to maintain chromosomal stability, which is essential for cell viability 4, 5. Our previous studies showed that the inhibition of telomere maintenance by telomerase inhibitors can inhibit the growth, migration and invasion of endometrial stromal cells derived from patients with endometriosis 24. In this study, we also found that the viability of endometrial epithelial cells and stromal cells derived from the endometria of endometriotic patients was significantly decreased with the treatment of telomerase inhibitor BIBR1532.

Although telomere-based cancer research has long been reported, the studies between telomere and endometriosis were lacking and inconsistent 25, 26. Sasamoto *et al.* 27 found that shorter telomere length in peripheral blood leukocytes was associated with an increased risk of endometriosis, and they believed that it is inflammation that contributes to telomere attrition. This is consistent with the findings of Gleason *et al.*, who found that leukocyte telomeres were 1% shorter every year since endometriosis diagnosis, accompanied by elevated C-reactive protein 28. However, both Bai C *et al.* 29 and Wang Y *et al.* 30 reported that the longer telomere length of peripheral blood leukocytes may increase the risk of endometriosis. Hapangama *et al.* 6 also reported that telomere length was significantly longer in the eutopic endometria of women with endometriosis, attributing the development of ectopic lesions to the increased telomere length in the eutopic endometria.

In this study, we collected 42 samples, including postmenopausal endometria, eutopic endometria and ectopic lesions in the proliferative and secretory phases, to assess the differences in telomere length between endometrial tissues in endometriotic patients and those in control women. We found that, compared with normal endometria, the longer telomere length of endometriotic patients' endometria can be detected in both the proliferative and secretory phases, whereas no significant increase in telomere length was found in ectopic lesions. Combined with Sampson's retrograde menstruation theory 31, which postulated that retrograde menstruation grows outside of the uterus and results in endometriosis, we suggest that the telomere maintenance variants in endometrial tissues of endometriotic patients may originate in the eutopic endometria. The higher telomere length of endometrial-tissue fragments in refluxed menstrual blood may promote endometrial cells survival and contribute to the formation of ectopic lesions, while longer telomere lengths are not necessarily required for the maintenance of ectopic lesions.

Telomere length maintenance is a complex process, aided by both telomerase-mediated pathways and the alternative lengthening of telomeres (ALT) pathways, with numerous genes involved 32. To uncover the underlying mechanisms of telomere maintenance for endometriosis development at the molecular level, we screened telomere maintenance genes from the Telnet database. To date, 2093 genes have been found to be involved in telomere maintenance, with 23727 edges constructed even when the interaction score was set to 0.7. The functions of the main clusters of the network were also analyzed, which mainly correlated with DNA and RNA.

Endometriosis is a hormone-dependent disease 1, 33. The progesterone resistance and estrogen dominance in the endometria of patients with endometriosis lead to the increased lesion growth and contribute to pelvic pain and infertility 1, 34. Androgen can be converted to estrogen by aromatase, thereby increasing the local estrogen levels 35. The roles of sex hormone receptors in endometriosis development were also investigated 36. In this study, the results showed that the telomere maintenance genes were significantly enriched in sex hormone-related pathways, including 5 estrogen-related pathways, 1 progesterone-related pathway, 1 androgen-related pathway, and 1 FSH-related pathway, with a total of 133 genes related to telomere maintenance. The genes, including FOSB, FOS, SRC, MAPK3, AR, MAPK1, and HSP90B1, which participate in telomere maintenance and sex hormone-related pathways, were also differentially expressed between eutopic endometria of endometriotic patients and normal endometria. However, the differential expression of these genes between ectopic lesions and their matched eutopic endometria derived from the same patients was not meaningful, suggesting that telomere maintenance may have more of an impact on the eutopic endometrium. This is also consistent with the results of telomere length detection.

Next, to test the diagnostic value of telomere maintenance, we screened five independent factors using LASSO regression, including EXO1, FOS, KDM4A, SMARCC1 and SULT1C2, among telomere maintenance genes. The results showed that there is a good predictive value of the model for endometriosis diagnosis, of which the AUC for the training dataset was 0.993 and the AUC for the test dataset was 0.883.

In this study, we have basically concluded that telomere maintenance may contribute to the development of endometriosis. And the gene sets that appear to have predictive value for endometriosis development were also identified. However, the roles of many telomere maintenance genes in the formation of endometriosis are unclear because of their ambiguous roles in endometriosis development and uncertain telomere maintenance functions. Some enhancing factors of telomere maintenance were decreased in the eutopic endometria of endometriotic patients, while some repressive factors were increased, such as SRC and MAPK1, which need to be further explored. Moreover, cyclic changes in the endometrium are a complex process involving alterations in hormonal and molecular signaling pathways. More precise grouping may also be required. Therefore, we need to collect more samples for further verification and to explore the underlying molecular mechanisms.

## Figures and Tables

**Table 1:**
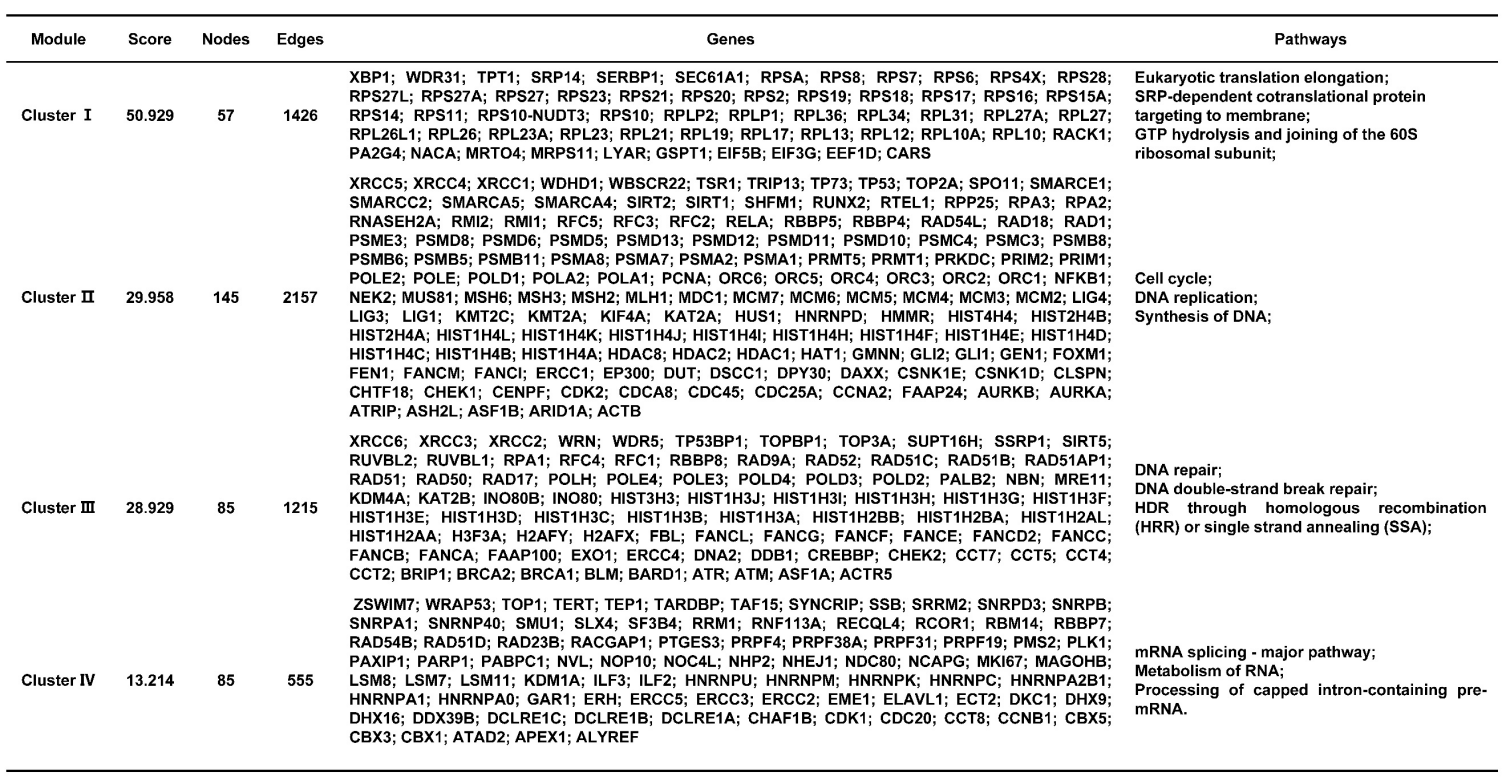
The information of 4 clusters scoring above 10.

**Table 2:**
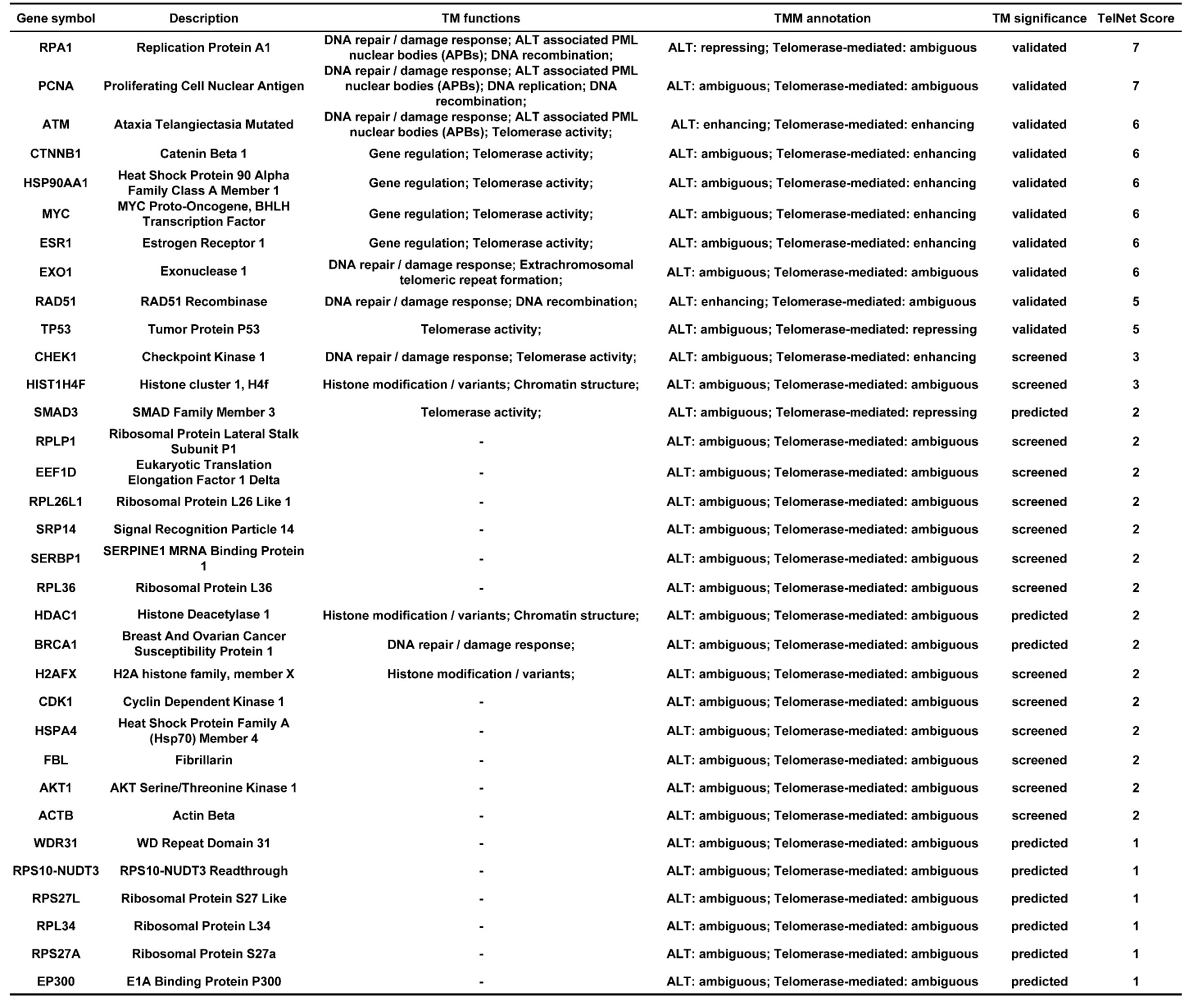
The information of 33 hub genes.

**Table 3:**
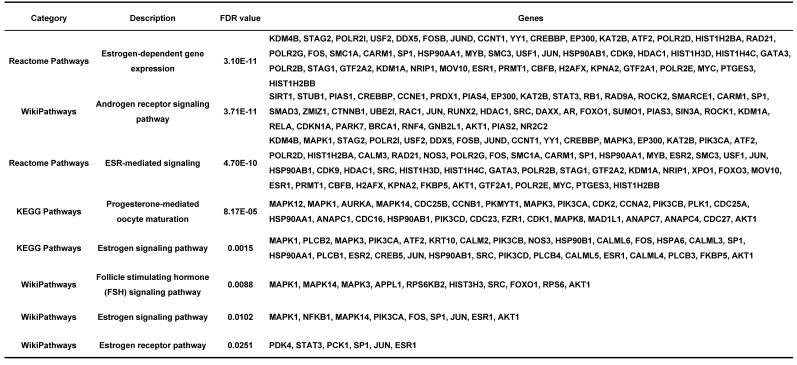
The enriched hormone-related pathways.

**Table 4:**
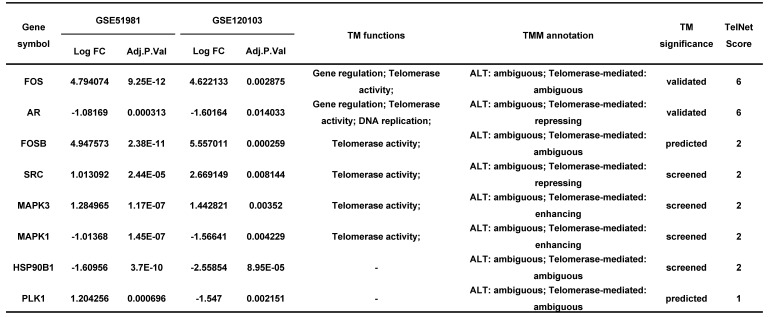
The information of significantly differentially expressed hormone-related genes in GSE51981 and GSE120103.

**Table 5:**
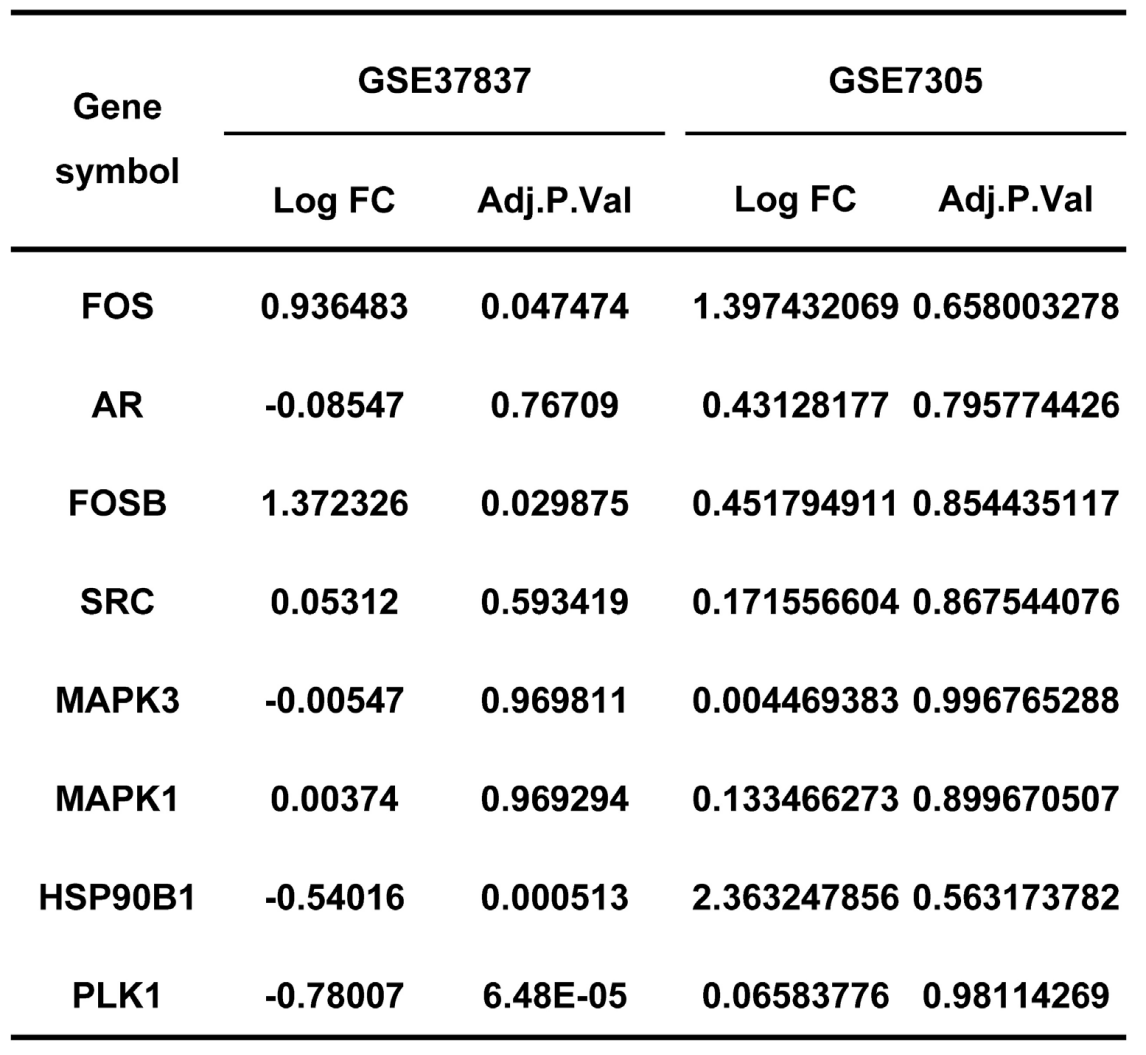
The expression of hormone-related genes in GSE37837 and GSE7305.

**Table 6:**
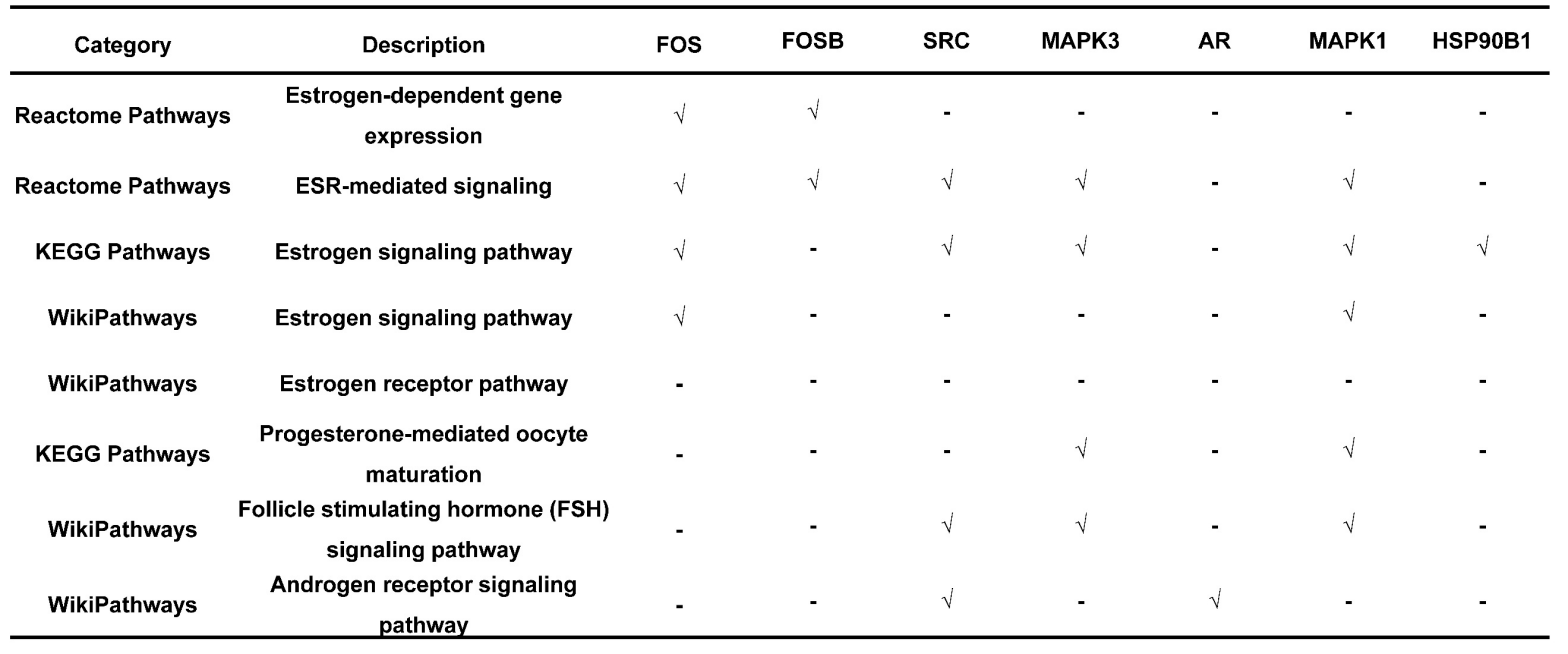
The telomere maintenance genes involved in steroid hormone signalings.

**Table 7:**
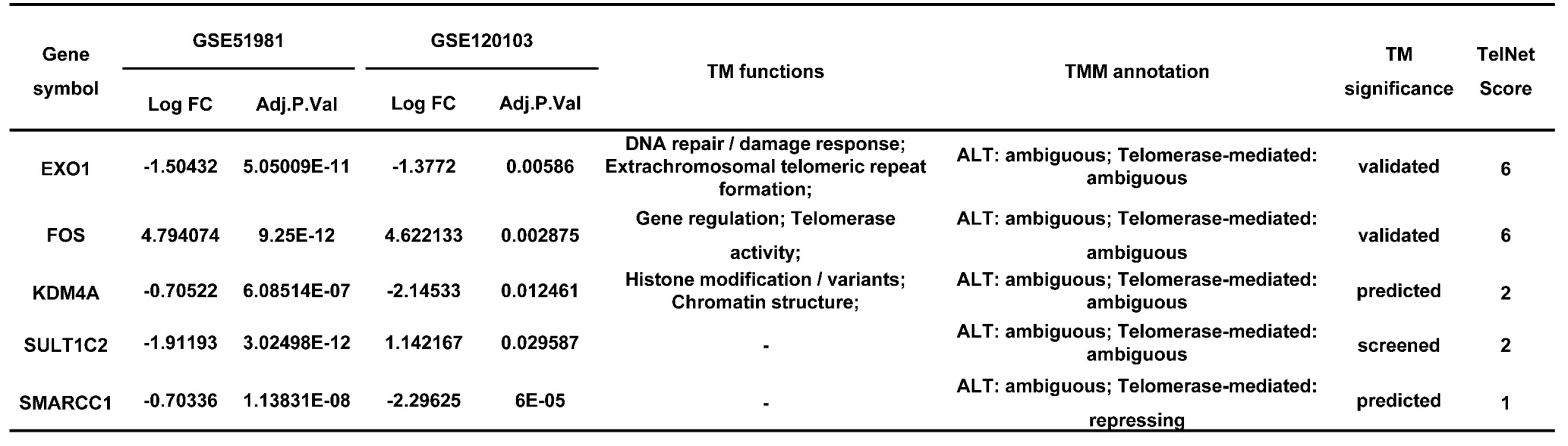
The information of diagnostic genes in GSE51981 and GSE120103.

**Table 8:**
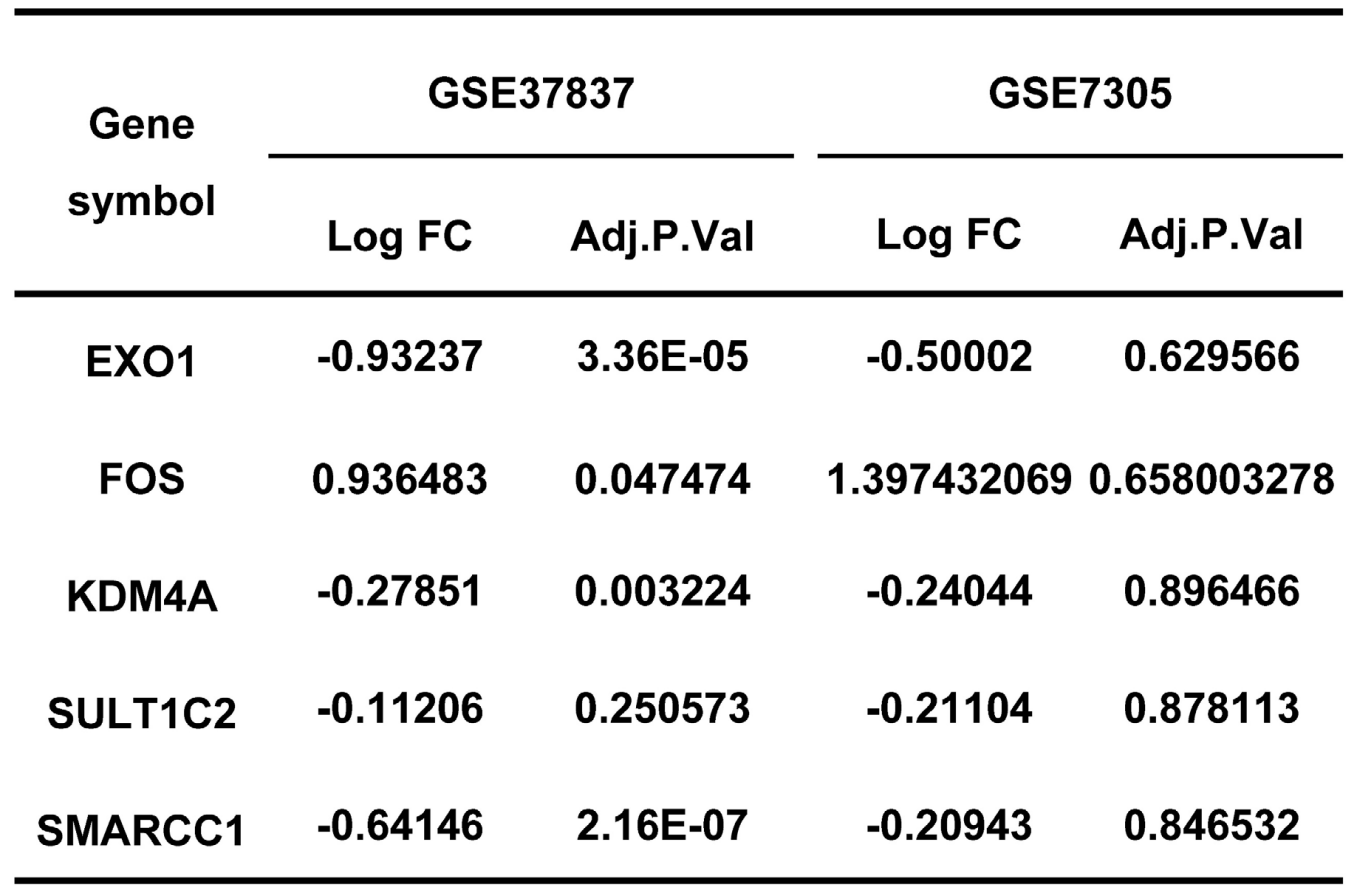
The expression of diagnostic genes in GSE37837 and GSE7305.
